# Numerical investigation of the impact of nonuniform corrosion on dynamic characteristics and nonlinear cyclic behaviour of circular RC bridge piers

**DOI:** 10.1007/s10518-025-02131-y

**Published:** 2025-03-07

**Authors:** Ziliang Zhang, Hammed O. Aminulai, William Powrie, Mohammad M. Kashani

**Affiliations:** University of Southampton, Southampton, UK

**Keywords:** RC bridge piers, Earthquake engineering, Nonuniform corrosion, Sledgehammer test, Cyclic degradation

## Abstract

Insufficient detail in the numerical modelling of reinforced concrete (RC) bridge piers can lead to oversimplification between simulated and real column behaviour under seismic loading. This paper describes the development and validation of an advanced and computationally efficient numerical model for circular RC bridge columns. First, the lateral stiffnesses, natural frequencies and damping ratios of three differently configured RC columns at various stages of degradation were evaluated by means of quasi-static cyclic and sledgehammer tests in loading cycles of increasing lateral drift amplitude. Normalised column lateral stiffness and first mode natural frequency were found to reduce nonlinearly with increasing column drift ratio. The two variables were also correlated to link RC column degradation with natural frequency reduction, which could allow rapid post-earthquake assessment of residual capacity. RC columns suffering from heavy corrosion were found to have a higher natural frequency and a tendency to fail prematurely under cyclic loading, whereas the damping ratio was generally unchanged. A set of nonlinear beam-element models employing fibre-discretised cross-sections was then developed and validated against experimental measurements. The model simulates buckling, fracturing, low-cycle fatigue, and bond-slip of vertical reinforcements, as well as nonuniform geometrical and mechanical deterioration of critical column sections. Individual fibre responses in the numerical model offered explanations for specific features of the experimental column stiffness and natural frequency reduction curves. Underlying mechanisms included the redistribution of compressive stress between concrete and rebars during cyclic loading, crushing of cover concrete, and yield of vertical reinforcements. Overall, the model accurately simulates the hysteresis response of the differently configured RC columns, without the need for column-specific adjustments.

## Introduction

### Background and context

The high economic cost of RC infrastructure deterioration is a world-wide problem, reported to consume up to 3.4% of global gross domestic product annually (Bowman et al. 2016). Understanding the seismic performance of ageing reinforced concrete (RC) bridge piers is important to owners and managers of bridges in earthquake-prone regions. The seismic capacity of RC bridges decreases as corrosion takes place, influencing especially the brittle collapse mechanisms that are strongly affected by bridge condition and maintenance (Crespi et al. 2020). Various attempts have been made to identify the detailed chemical mechanisms for advanced RC deterioration models (Andisheh et al. 2016), the influence of the key characteristics of RC columns on model parameters (Liu et al. 2023), and from a broader perspective the overall effect of ageing on the seismic performance of RC bridge piers evaluated using probabilistic tools (Choe et al. 2008).

Predicting the seismic or cyclic response of RC bridge columns, especially ageing ones, is complicated in several ways. Evidence in the literature has shown that corrosion creates nonuniformity of cross-section along the length of reinforcing bars (rebars), with detrimental effects on their ultimate strain, ductile area, and elongation capabilities (Du et al. 2005a), and tends to reduce the overall force-bearing capacity of the RC column more rapidly than an assumed uniformly corroded scenario (Du et al. 2005b; Pelle et al. 2022). Potential buckling of vertical reinforcements in RC columns may also affect the gradual degradation and capacity loss of columns subjected to cyclic loading (Lavorato et al. 2020). The key parameter governing the buckling stability of a vertical reinforcement in compression is its buckling length, which influences the fracture (El-Bahy et al. 1999) and pinching (Kashani et al. 2016a) characteristics of RC columns. Another important aspect regarding the seismic capacity of RC bridge columns is their low-cycle fatigue degradation when subjected to seismic loading, which has often been neglected in previous studies (Berto et al. 2009; Alipour et al. 2011). It has been shown that monotonic pushover analyses, which do not consider the low-cycle, high-amplitude fatigue of RC columns, are inadequate for evaluating their seismic capacities (Kashani et al. 2016b). Recent experimental and numerical studies on standalone reinforcement bars (Fernandez et al. 2016) and aged RC columns (Li et al. 2015) have also highlighted the impact of low-cycle fatigue on the response of corroded reinforcement bars. Two key parameters that are affected by, and can be used to quantify, corrosion degradation of an RC column are the natural frequency and the stiffness. While these two parameters are intrinsically correlated, likely nonlinearly, the former is more readily measurable on-site, whereas to measure the latter is much harder.

Currently, numerical modelling of ageing RC bridge piers rarely takes all abovementioned phenomena into account. Ageing RC columns in bridge models are usually represented using macro elements (for example, a single spring element with a phenomenological hysteresis model), with approximated properties for ageing. Even where columns are modelled explicitly, the effect of ageing is often represented by a uniform reduction of cross-sectional areas (Biswas et al. 2022). Such models thus do not incorporate the structural and material detail unique to each RC bridge column.

This lack of detail in modelling the effects of material and structural degradation leads to discrepancies between simulated and true column and bridge behaviours in cyclic loading. There is a need to replace the commonly adopted simplified modelling techniques to those that can adequately reflect the ageing of RC bridge piers, as verified by direct physical evidence. Unfortunately, real-world data from RC bridge columns with nonuniform corrosion effects with which to calibrate and validate analytical or numerical approaches can be hard to find (Abtahi et al. 2023). The literature is also lacking in recommendations on appropriate modelling techniques for implementing sub-element level details in finite-element models employing fibre-discretised cross sections (Menegotto and Pinto 1973), as well as measures to overcome numerical instabilities or convergence issues.

Understanding the effect of cyclic loading on the evolution of natural frequency and stiffnesses of degraded RC bridge piers can also be useful in the rapid post-earthquake structural assessment of RC bridges (Ge et al. 2021; Alexander et al. 2023). The reduction of RC bridge pier structural capacity is the result of its hysteretic response to strong ground motions, as reflected, to a degree, by its residual stiffness. Knowledge of the residual stiffness allows informed decisions to be made when assessing damage sustained after an earthquake. Residual stiffnesses of RC bridges and structural members are often not realistically obtainable on-site or for large numbers of bridges. Information regarding the correlation between pier stiffness and another structural state variable, which can be measured with relative ease in the field, thus provides a useful reference to support post-earthquake field investigations. It is therefore of interest to correlate the normalised residual stiffness to the percentage change in natural frequency. The original natural frequency of a (structural member of a) bridge can readily be obtained from design documentation, or reassessed by means of modal analysis on a finite-element model. The residual natural frequency can be obtained by means of ambient (Brownjohn et al. 2010) or forced vibration testing (Severn et al. 1980).

### Research contribution and novelty

In response to the knowledge gaps identified, the first objective of the research reported in this paper was to investigate experimentally the evolution of lateral stiffness, natural frequency, and damping ratio of brand-new and ageing RC bridge piers subjected to repeated quasi-static loading cycles with monotonically increasing lateral displacement amplitudes, by performing a set of large-scale laboratory sledgehammer and cyclic tests on three differently configured circular RC bridge column specimens.

The experimental observations were then used to develop and validate a set of advanced, computationally efficient nonlinear beam element, fibre section finite-element models, with sufficient detail to accurately predict both the cyclic response and evolution of the dynamic characteristics of ageing RC bridge columns. These models represent explicitly the nonuniform geometrical and mechanical deterioration of steel reinforcements due to corrosion, and the effects of inelastic buckling, low-cycle fatigue, bond slip and rebar fracturing, the precise as-constructed locations of vertical reinforcement bars, and corrosion on unconfined and confined concrete materials. The models were then used to fulfil the second objective of the research; to replicate numerically the hysteresis responses and degradation histories of the experimentally tested RC columns.

##  Laboratory Cyclic and sledgehammer tests of ageing Rc Bridge columns

### Experimental testing scheme

Three RC columns specimens, one uncorroded (Column A) and two corroded (Column A1 and Column B1) were tested in the UKCRIC National Infrastructure Laboratory (NIL) at the University of Southampton, UK. Experimentally, the paper focuses primarily on the evolution of the dynamic properties of RC bridge columns with respect to repeated loading cycles, in which the displacement amplitude increases monotonically. Experimental aspects concerning the preparation of the full experimental campaign, detailed examination on the state of corrosion of the specimens, as well as investigations regarding the nonlinear quasi-static hysteresis behaviours of RC columns, are detailed in (Kashani et al. 2024).

**Fig. 1:**
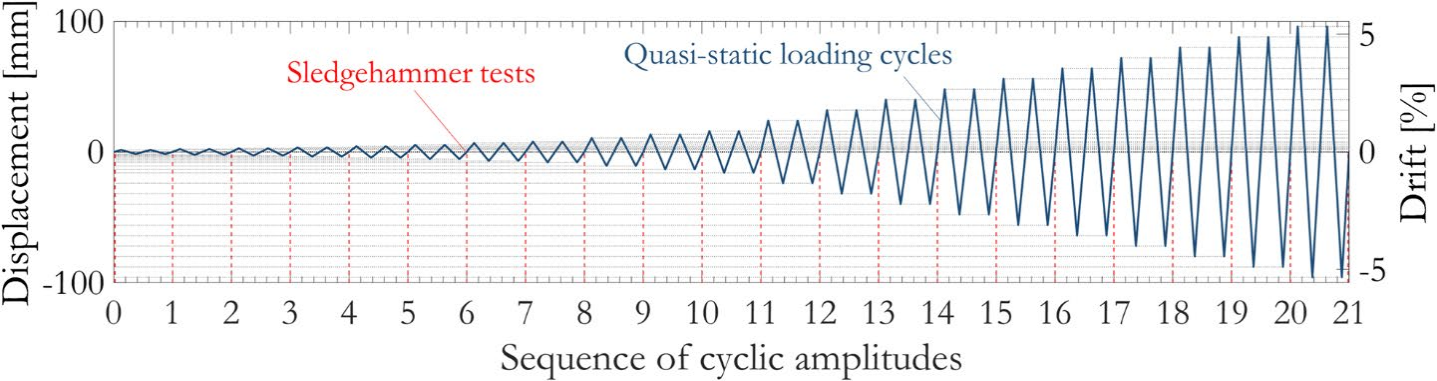
Cyclic loading protocol and sledgehammer testing scheme

**Fig. 2:**
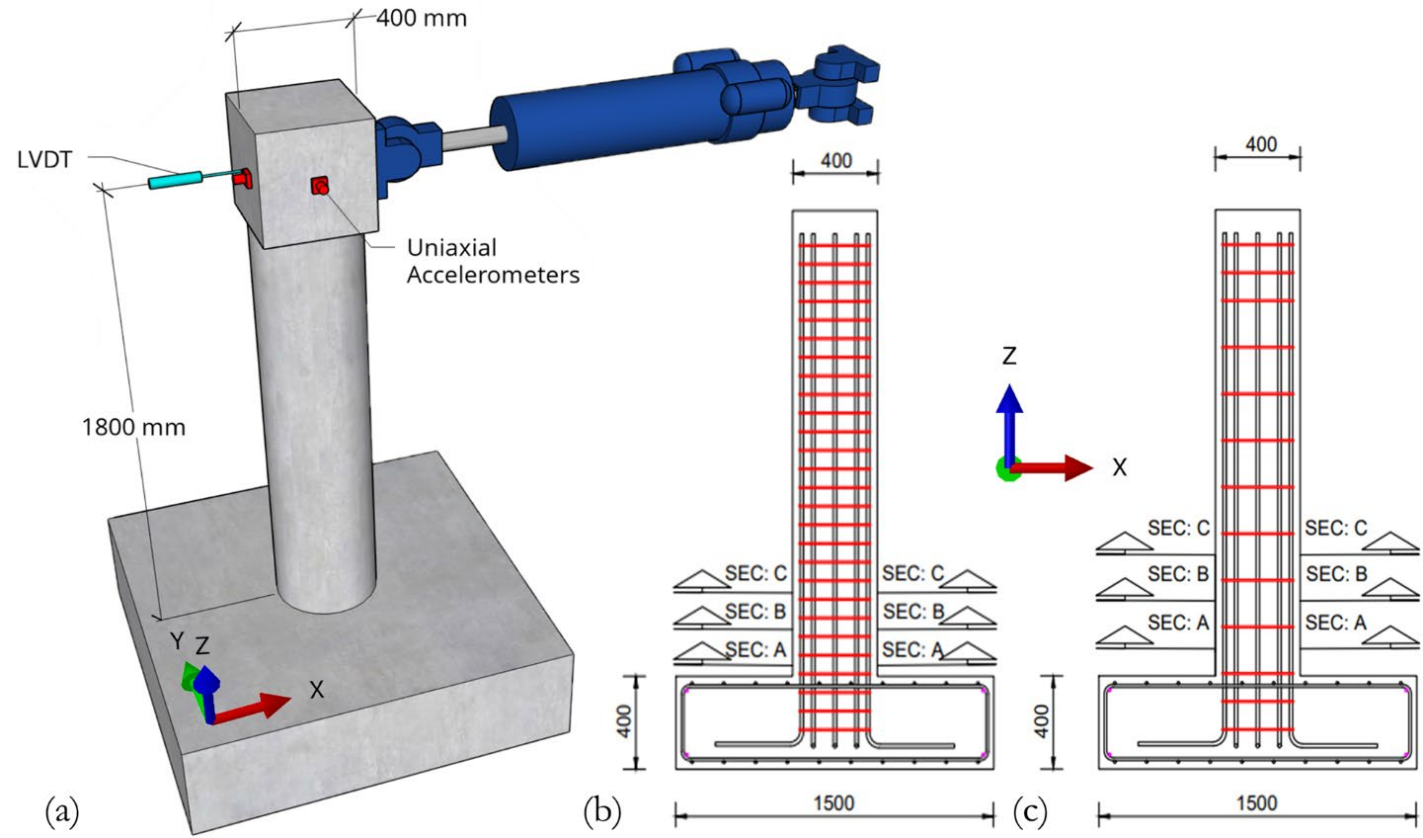
Diagrams of the experimental test setup: **a** overview of the specimen and instrumentation, **b** rebar configuration for Column A and Column A1, and **c** rebar configuration for Column B1. *LVDT* linear variable differential transformer (displacement transducer), *SEC* cross-section

The columns were subjected to a specific sequence of cyclic and sledgehammer tests as shown in Fig. 1. In total, 21 levels of displacement amplitude were prescribed ranging from 1.6 mm to 96.0 mm, corresponding to a column drift ratio of up to 5.3%. Two loading cycles were applied per displacement amplitude level, as recommended in (ACI Committee 374 2013). The loading cycles were applied via a 250 kN force capacity, 250 mm stroke MTS actuator under displacement control. The actuator is equipped with a built-in load cell and a displacement transducer (LVDT) on the opposite side of the actuator mounting point (Fig. 2), to monitor the force-displacement response at the top of the column. The columns were not subjected to any other external loading, which is justified by zero or low levels of axial force typically adopted in similar existing tests for bridge columns in the literature. As summarised in (Kashani et al. 2016a), the level of axial force used in previous experiments of standalone RC bridge piers is generally between 0 and 12%; and zero axial load, while admittedly situtes at the lower end of that range, has indeed been adopted in previous large-scale RC column tests such as in (Hamilton et al. 2003). It has been shown numerically that high degrees of variation (10 to 30%) in axial load could lead to changes in RC bridge column esponse, yet such changes were reflected only in terms of different response magnitudes and did not involve a different failure mode (Dizaj et al. 2018). To avoid catastrophic failure of the specimens in the laboratory, cyclic loading scheme was terminated if the test specimen showed obvious signs of distress, for example significant concrete spalling or crushing, rebar buckling or bucketing, or multiple rebar fractures.

Sledgehammer tests were carried out before commencing the sequence of static loading cycles and after the completion of each level of cyclic displacement amplitude, as indicated by the red dashed lines in Fig. 1. The columns were struck by a sledgehammer equipped with a load cell, along directions parallel (x-axis) and perpendicular (y-axis) to the direction of cyclic loading. Five sledgehammer blows were performed for each cyclic testing amplitude and strike direction, and averaged estimates of the natural frequency and damping in both time and frequency domains were determined. Natural vibrations of the RC column specimens were measured by two uniaxial accelerometers installed on two perpendicular faces at the elevation of the actuator mounting point (Fig. 2).

The sledgehammer tests before and after each amplitude of cyclic load were carried out with the RC columns in their cyclic testing configuration (i.e., connected to the actuator). The connection restrains x-axis motions at the top of the column and may also affect swaying stiffness of the column-actuator system in the y-direction. The magnitude of the additional stiffness introduced by the actuator can be difficult to estimate considering the complex constraint details. The connected actuator also introduces considerable additional mass to the overall column-actuator system, the amount of participating mass likely depends on the connection details. Preliminary analyses suggested that the influence of the additional mass was far greater than the additional stiffness introduced by the connecting actuator, meaning that the natural frequencies of the tested RC columns under the free-standing condition would have been much higher than those measured under the cyclic testing configuration. This is a compromise that was considered necessary from a practical perspective. It was preferable not to detach the actuator after every quasi-static loading cycle to carry out sledgehammer tests in strictly free-standing conditions, to avoid the potential for disturbing the experimental setup to the detriment of subsequent cycle loading data. In any case, the influence of the column boundary condition can be negated by examining the evolution of the normalised, rather than the absolute, natural frequencies with respect to the sequence of quasi-static loading cycles. In addition, freestanding sledgehammer tests were carried out on column specimens A1 and B1 before and after accelerated corrosion. For investigating the effect of corrosion on column natural frequencies, the columns were under a free-standing condition but were always placed on wooden pallets. The pallets were practically necessary for the subsequent lifting of the specimens using an industrial forklift. Hence, the measured values of natural frequency might be different to a condition where the specimens are placed directly on the ground. Nevertheless, the normalised difference can still reflect the influence of corrosion. The potentially possible but improbable degradation of the wooden pallets reduce the overall stiffness of the specimen-pallet systems.

In the context of the present study, accelerated corrosion refers to artificially induced metallic corrosion at a much faster rate than the corresponding natural process, allowing the effects of RC column corrosion such as rebar cross-section loss and the deterioration of rebar and concrete material properties to be reproduced in the laboratory (Kashani et al. 2024). The external current method, previously implemented in experimental campaigns investigating similar RC columns (Ge et al. 2020; Aminulai et al. 2023a, b), was adopted to artificially deteriorate column specimens A1 and B1. Corrosion is induced by applying an electrochemical potential between an anode (i.e., the metal to be corroded, in this case the rebars embedded within the RC columns) and a cathode (stainless steel plates), with both immersed in a 5% sodium chloride (NaCl) saline solution. The electrolyte solution was contained in bespoke hollow cylinder vessels, each was attached and sealed against the upper face of column footing, and the liquid submerged approximately the lower half of each column. The induced electric current was applied via cables connected to the vertical reinforcement bars from the very top of each column, of which the magnitude was controlled to be approximately 5 A and the corrosion process took approximately eight weeks to complete on Column A1 and six weeks for Column B1. Horizontal and vertical cracks were then apparent on the surfaces of the corroded RC column specimens. This can be attributed to the accumulation of corrosion product between the rebar and the surrounding concrete causing an internal volumetric expansion. These corrosion cracks appeared to distribute relatively evenly over the corroded area. No axial load was applied on the specimens during accelerated corrosion. Note that, in practice, the accelerated corrosion process can be also dependent on various other factors, including: the size and shape of anode (rebars) and cathode, the actual exposure area of the electrodes (empirically the cathodes, on which corrosion products can often accumulate rapidly), natural shifts of the actual applied current intensity, the relative nobility of the two electrode materials, and most importantly the electric connectivity of all rebars within the concrete specimen (in case when the tested EC specimens have complex rebar arrangements). It is therefore that an effective current density value (Nguyen and Lambert 2018) might be difficult to meaningfully determine for large RC specimens such as the columns herein. Instead, the actual corrosion mass losses were measured physically after the completion of all cyclic and sledgehammer tests.

### Specimen specifications

**Fig. 3:**
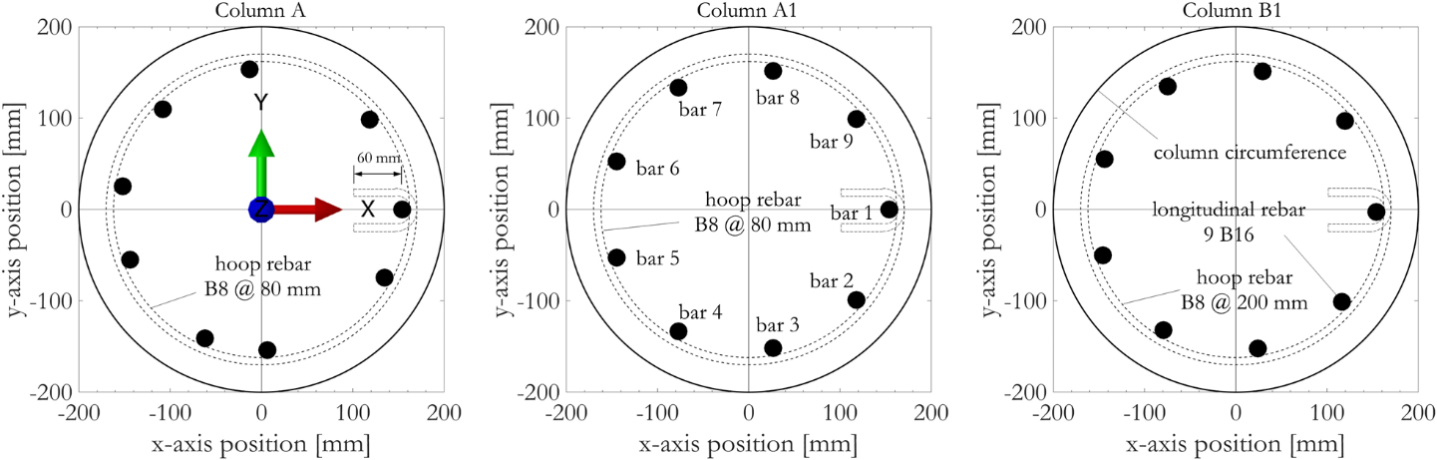
Cross-sectional locations of vertical reinforcement bars in the three RC column specimens. Quasi-static actuator forces are applied along the x-axis during cyclic testing

Specifications of the three circular RC columns specimens are summarised in Table 1 (Kashani et al. 2024). All three columns were designed to Eurocode 2 (CEN 2004a) targeting the same flexural capacity, and their aspect ratio is considered representative based on the PEER Report 1998/01 (Lehman and Moehle 2000). They therefore have the same nominal properties for longitudinal rebars as well as concrete material. Concrete compressive strength for each column was measured directly from the specimens, the differences were aleatory. Columns A and A1 represent modern RC bridge piers that are also designed for earthquake resistance according to Eurocode 8 (CEN 2004b), with a hoop rebar spacing of 80 mm for increased shear capacity and ductility. Column B1 represents non-seismically designed, older generation bridge piers with lighter concrete confinement, with a hoop rebar spacing of 200 mm. The exact positions of the nine individual vertical reinforcement bars within the cross-sections of the three specimens, examined after completion of the tests, are shown in Fig. 3. Column A differs from the other two specimens in that its longitudinal reinforcement bars are unevenly distributed, owing to construction errors during specimen preparation. Longitudinal rebars in the cross-sections of both Column A1 and Column B1 were spaced evenly and distributed symmetrically with respect to the x-axis. In terms of deterioration, Column A was never subjected to accelerated corrosion, whereas Columns A1 and B1 were. The authors hereby highlight the uncertainties associated with specimen construction, accelerated corrosion implementation and the limited number of test specimens. They underline the fact that the tested specimens do not relate to any real-world bridge pier prototype. They also stress that probable failure modes between circular and rectangular columns can be very different due to the different geometries, so that this study does not assume relevance to the latter.

The actual corrosion condition of longitudinal and hoop rebars at three evenly spaced column segments located near the bottom of Column A1 and Column B1 was measured in terms of percentage mass losses, after completion of all cyclic and sledgehammer tests (Table 2). Corrosion was greater in Column B1. Such measurements are important because nonlinear responses to lateral forces (for example, during earthquakes), leading to plastic hinge formation, tend to be concentrated within a limited length near the end of an RC bridge piers. It is as yet unknown whether the neglect of nonuniformity in rebar corrosion distribution, for example by modelling plastic hinges using macro elements, would result in inaccurate or unsafe structural performance estimates in numerical analyses. The central cross-section of each segment is denoted in Fig. 2 as A, B, and C. For Column A1, the length of each segment was twice the hoop rebar spacing, i.e., 160 mm. For Column B1, the segmental length was 200 mm, corresponding to one hoop rebar spacing. These values were selected to reflect the experimentally observed variation of corrosion along the length of longitudinal rebars with reasonable resolution (Kashani et al. 2013a), while keeping the practical and subsequent numerical implementation challenges manageable. A total of 27 longitudinal rebar segments and 6 or 3 hoop rebars, for Columns A1 or B1 respectively, were cut from each column specimen, with measurements taken after rust residue on the dismembered rebars had been removed. Corrosion in Column B1 was found to be slightly more severe than in Column A1. In terms of corrosion distribution, the most corroded segment among the three for Column A1 was Segment A, located at the very bottom. For Column B1, the middle Segment B was found to have suffered the greatest corrosion mass loss. Data of nonuniform rebar corrosion mass loss is used later in this paper for the development and validation of numerical models.

**Table 1:** Specifications of the three tested reinforced concrete (RC) columns

Properties	Column A	Column A1	Column B1
Circular cross-section diameter [mm]	400		
Column length (top-of-foundation to actuator mounting point) [mm]	1800		
Nominal longitudinal rebar diameter [mm]	16		
Nominal hoop rebar diameter [mm]	8		
Concrete cover thickness [mm]	30		
Rebar Young’s modulus [GPa]	200		
Rebar yield strength [MPa] and strain [%]	530 MPa at 0.23%		
Rebar ultimate tensile strength [MPa] and strain [%]	630 MPa at 12%		
Rebar fracture strain [%]	21%		
Measured 28 days cylindrical concrete compressive strength [MPa]	60.3	59.0	50.1
Hoop reinforcement spacing [mm]	80	80	200
Longitudinal rebar distribution (see Fig. 3)	Unevenly spaced (construction error)	Evenly spaced	Evenly spaced
Subjected to accelerated corrosion	No	Yes	Yes

**Table 2:**
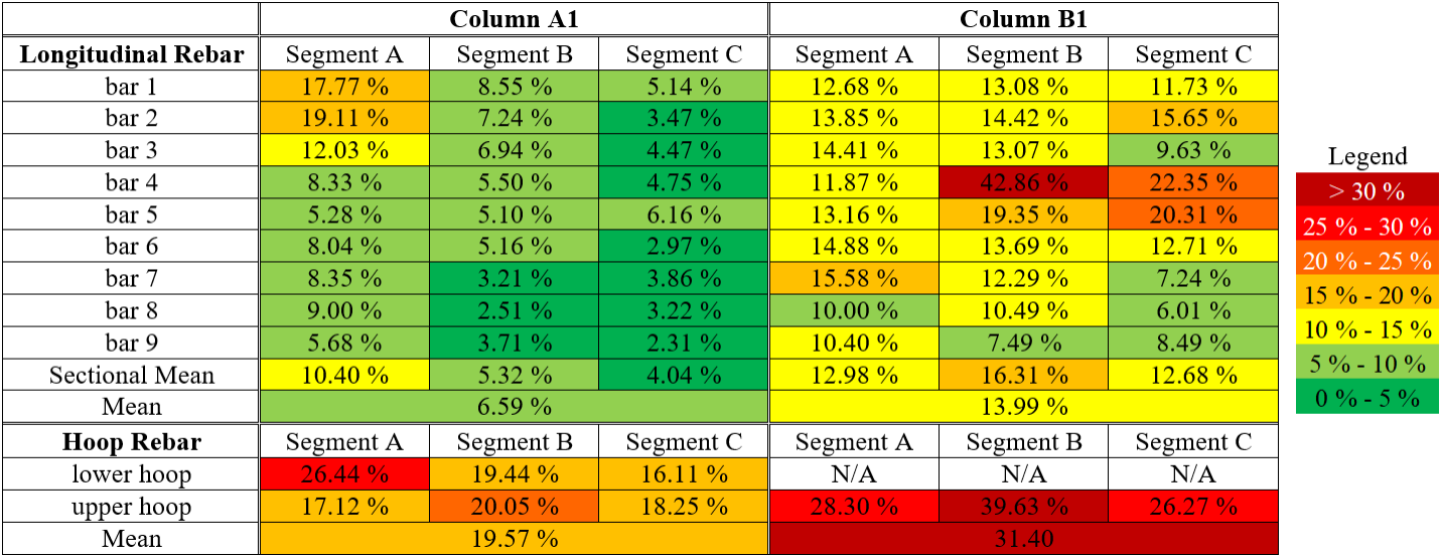
Corrosion induced rebar percentage mass losses measured on three critical column segments near the bottom of column A1 and column B1. The centre of each Segment corresponds to the three Sections indicated

### Evaluation of natural frequency of vibration and damping ratio

#### First mode natural frequency of vibration

The first mode natural frequencies of the RC column specimens were identified in the frequency domain by examining windowed and averaged power spectra of the recorded x- and y-axis acceleration responses. Time domain responses were truncated, zeroed, and converted to the frequency domain by means of a Fast Fourier Transform (FFT). The Fourier amplitude spectra were then windowed using a 64-point Chebyshev window (MathWorks Inc. 2023), which facilitates the identification of and distinction between closely located frequency response peaks. To reduce random noise, FFTs were computed and filtered separately for each of the acceleration time history responses from the five sledgehammer hits, and the ensemble averaged. Power spectral density functions, or power spectra, were then estimated as the square of the magnitude of the averaged FFT spectral amplitudes. This further distinguishes spectral peak responses from ambient noise and from each other.

Identification of first mode natural frequency was automated using a script written in Matlab (MathWorks Inc. 2023) to identify the highest peak in the power spectrum within a probable frequency range, determined as 5 to 25 Hz from preliminary analyses of the test results from the RC column specimens. This was to prevent interference from noise and higher mode responses that are known to be irrelevant. Anomalous cases, for example, when no absolute maximum value can be found or when multiple equally valued absolute maxima are detected within the specified frequency range, were flagged for manual inspection.

#### Damping ratio

The damping ratio was evaluated using two approaches: the half-power bandwidth method and the logarithmic decrement method (Chopra 2012). The half-power bandwidth method is a frequency domain approach, performed on raw or curve fitted frequency response function data, which estimates the damping ratio using the half-power bandwidth of the frequency response curve:

1 $$\:\xi\:=\frac{{f}_{2}-{f}_{1}}{2{f}_{n}}$$

where *ξ* is the damping ratio; *f*_*1*_ and *f*_*2*_ are the frequencies at which the power amplitudes are half of the maximum and are positioned to the left- and right-hand-sides of the peak respectively, and *f*_*n*_ is the estimated first mode natural frequency of the RC column specimens. The formulation implies that the half-power bandwidth method, in theory, requires a clearly distinguishable spectral response peak to have an accurate damping ratio estimation. In cases where multiple peaks overlap in the frequency response curve such that the half-power bandwidth of the highest peak cannot be directly determined, regression analysis was performed to fit a second order polynomial function to the data points that belong to the highest peak. The fitted function was then extrapolated to approximate the half-power bandwidth and to estimate the corresponding *f*_*1*_ and *f*_*2*_ values.

The logarithmic decrement method calculates the damping ratio of a single degree-of-freedom (SDOF) system by evaluating the logarithmic decay of its free vibration amplitude in the time domain. The key restriction to the use of this method is that the theory is only strictly applicable to SDOF systems, so that the estimation is inaccurate if coupled modes or multiple modes with closely valued natural frequencies are excited during the sledgehammer test.

In this study, a regression analysis procedure was incorporated into the logarithmic decrement method to reduce the impact of possible higher mode responses. It is known that the response amplitude of damped free vibration of an SDOF system can be written as:

2 $$\:A\left(t\right)={p}_{1}{e}^{-2\pi\:\xi\:\cdot\:{f}_{n}\cdot\:t}={{p}_{1}e}^{{p}_{2}\cdot\:t}$$

3 $$\:\xi\:=\frac{{p}_{2}}{-2\pi\:\cdot\:{f}_{n}}$$

where *A*(*t*) is the amplitude (envelope) of the damped free vibration of an SDOF system, as a function of time *t*; *p*_*1*_ and *p*_*2*_ are first order exponential function coefficients to be estimated by performing a regression analysis; *e* ≈ 2.718 is the base of natural logarithms; *ξ* is the damping ratio; and *f*_*n*_ is the known first mode natural frequency of the system, in this case the RC column specimens. For deriving a time domain estimation of the damping ratio *ξ*, each recorded acceleration time history is first zeroed and truncated at the front to remove the portion when the time series is still distinctly characterised by forced vibration or coupled natural modes. The truncated time series is set to start from three times the natural period after the moment of applying the sledgehammer blow, which is then filtered using an 8 pole, 5–25 Hz band pass Butterworth filter to reduce frequency contents known to be irrelevant to the first mode response. The filtered acceleration time history is then divided into multiple segments, each of which has the same duration as the estimated first mode natural period, 1/*f*_*n*_. Within each segment of time history, the absolute maximum acceleration value is determined. This procedure produces a set of data that represents the decaying amplitude envelope of the damped free vibration, *A*(*t*). A regression analysis can then be performed in which a first order exponential function is fitted and the regression coefficient *p*_*2*_ estimated to calculate the damping ratio *ξ*. Instead of considering only two response peaks spaced several cycles apart, this approach makes use of information contained throughout the full duration of the decaying motion, and the regression process essentially acts as an additional filter that further reduces the influence of higher mode responses.

##  Experimental results

### Transient responses of RC Bridge columns subjected to longitudinal and transverse sledgehammer hits

**Fig. 4:**
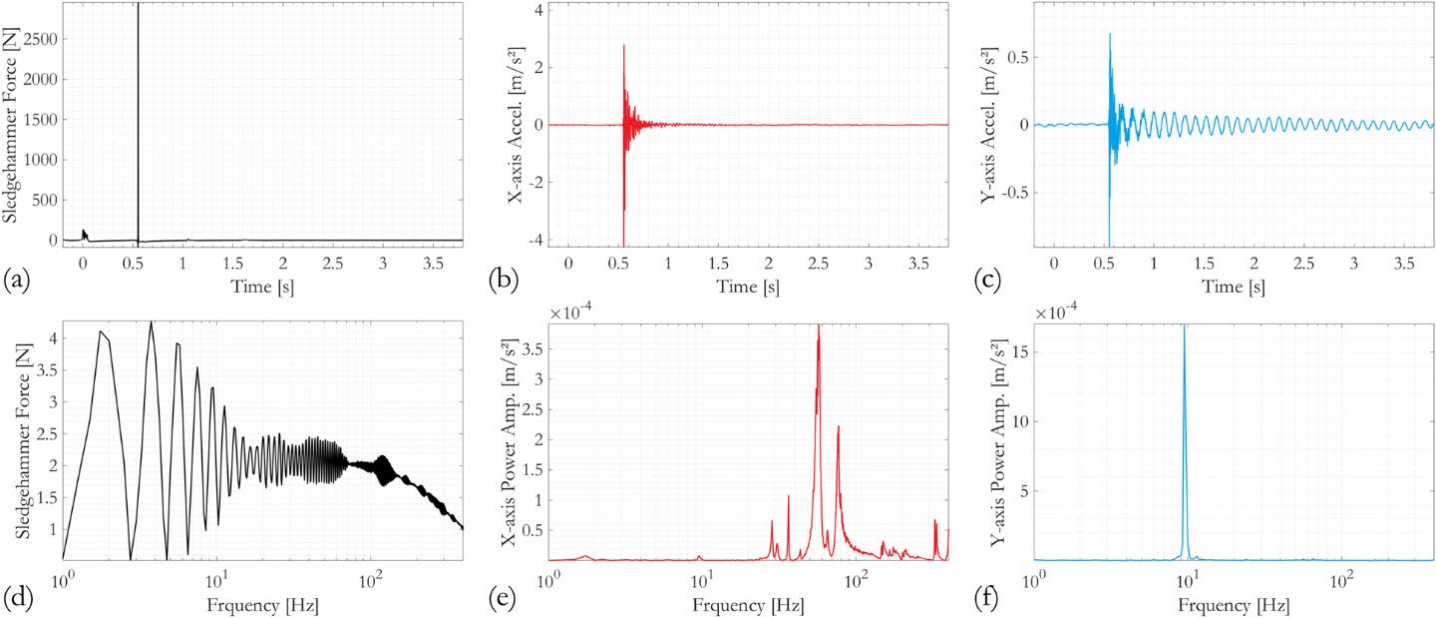
Measured transient responses from sledgehammer test performed on Column A after the completion of the cyclic testing sequence, where the sledgehammer force is applied on the x-direction, **a**–**c** time series; **d**–**f** power spectra. *Accel.* acceleration, *Amp.* amplitude

**Fig. 5:**
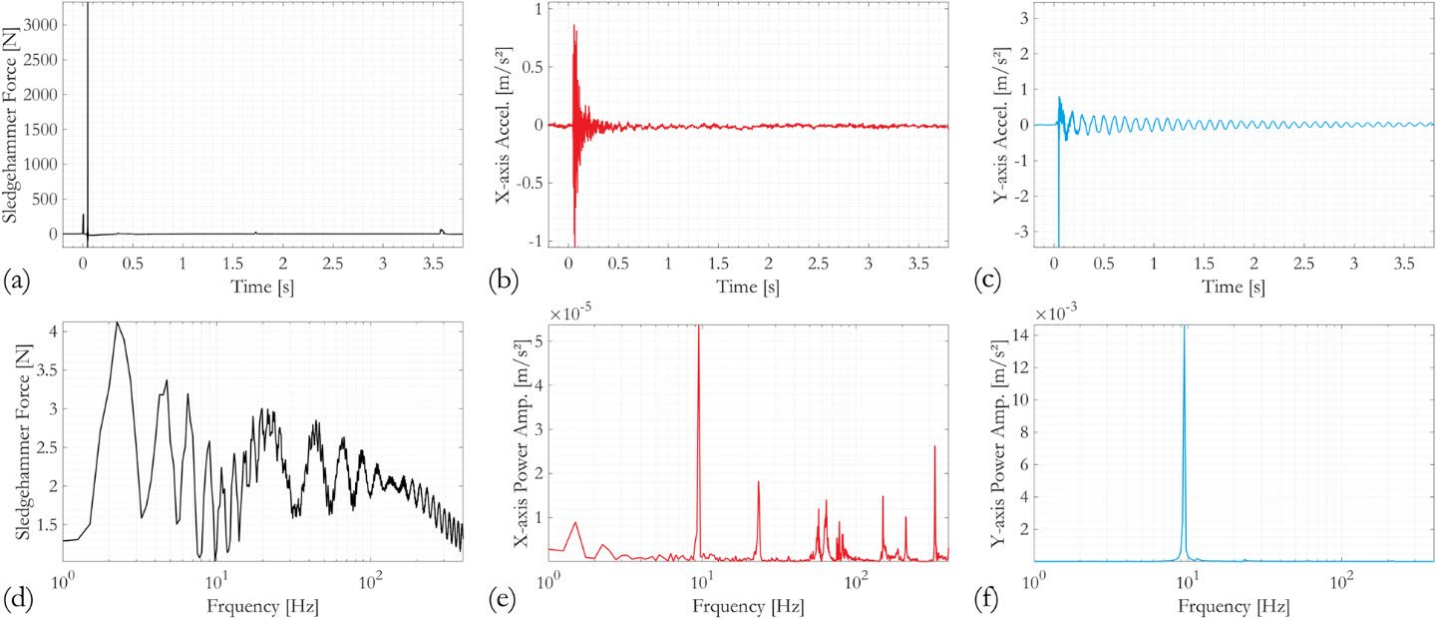
Measured transient responses from sledgehammer test performed on Column A after the completion of the cyclic testing sequence, where the sledgehammer force is applied on the y-direction, **a**–**c** time series; **d**–**f** power spectra. *Accel.* acceleration, *Amp.* amplitude

Exemplar results obtained during the sledgehammer test on Column A after the completion of the full cyclic testing regime are shown in Figs. 4 and 5, for sledgehammer strikes in the longitudinal (x-axis) and the transverse (y-axis) directions, respectively. Both the sledgehammer forces measured by the load cell and the acceleration responses measured by uniaxial accelerometers in the x- and the y-directions are examined in the time and frequency domains. The forces applied by the sledgehammer have magnitudes in the order of 2 × 10^3^ to 3 × 10^3^ N. The impact forces generally have short duration, on average 3 × 10^−3^ s, and their frequency contents are often found to be distributed relatively evenly over a wide range, approximately 1 to 150 Hz. These observations confirm that the measured time- and frequency-domain responses of the RC column specimens are dominated by their free vibrations.

The y-axis acceleration time history responses following sledgehammer blows in both directions (Figs. 4c and 5c) are predominated by vibrations of lower frequency than the x-axis responses (Figs. 4b and 5b). This observation can be confirmed by comparing the corresponding power spectra. It is further noted that the frequency domain responses in the x-direction differ significantly depending on the directionality of the sledgehammer blow (Figs. 4e and 5e), whereas the y-direction spectra appear similar (Figs. 4f and 5f). This implies that the fundamental mode of the RC column in its cyclic testing configuration, which is the cantilever-like sway of the column in the y-direction, is easily triggered by the sledgehammer blows regardless of their direction. The fundamental frequency is around 10 Hz. In contrast, the x-direction vibrations are higher modes, owing to the restraint imposed by the actuator. These x-axis modes are not as easily excited if the sledgehammer blow is applied in the y-direction, such that the highest response peak in the x-axis power spectrum (Fig. 5e) matches that in its y-axis counterpart at a frequency of approximately 10 Hz (Fig. 5f). In contrast, when the sledgehammer blow is applied in the x-direction, the 10 Hz peak becomes almost visually unnoticeable (although identifiable numerically) as the x-direction vibration modes of the specimen dominate the overall response. The most dominant is indicated by the highest response peak, just below 60 Hz (Fig. 4e) for this case.

### Effects of corrosion on the dynamic properties of RC Bridge columns

#### Natural frequency

**Fig. 6:**
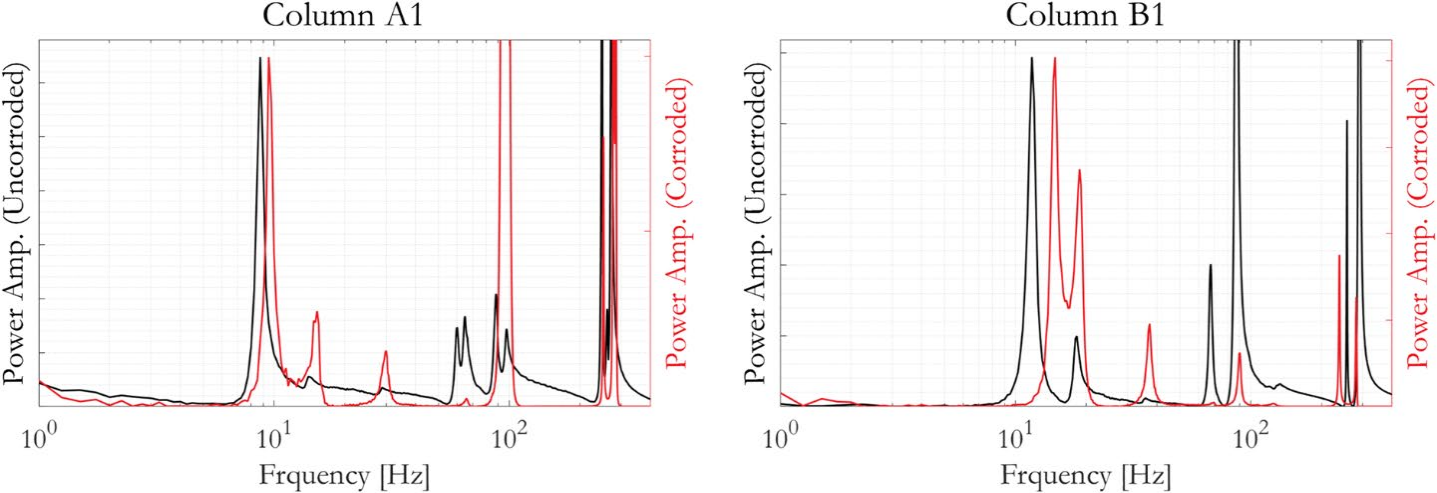
Comparison of y-axis power spectra before and after accelerated corrosion, from y-axis sledgehammer blows on specimens Column A1 and Column B1, normalised to the amplitude of the first mode response

Figure 6 compares the power spectra responses of specimens Column A1 and Column B1 before and after accelerated corrosion (all before the cyclic tests). The power spectra shown are y-axis responses to y-direction sledgehammer excitation. The fundamental natural frequencies for the uncorroded, free-standing (placed on shallow wooden pallets for lifting purposes later) specimens Column A1 and Column B1 were 8.5 Hz and 12.0 Hz, respectively. The mode shape corresponding to these natural frequencies is y-direction cantilever-like sway. Given that both column specimens were designed to have the same flexural capacity, their fundamental frequencies are supposedly similar. The minor difference observed is likely to be attributable to factors such as minor variations in the material and geometrical properties of concrete and steel reinforcements, as well as differences in the construction process. Nonetheless, the same trend is seen in both specimens when their responses before and after accelerated corrosion are compared. The presence of corrosion increased the first mode natural frequency of both columns A1 and B1. This may have been due to the formation of corrosion products, which has much lower density as compared to the original steel material. This internal volume gain, therefore, could fill in small gaps and/or increase contact pressure at the reinforcement-concrete interface, hence results in increased friction and bond. Via these local mechanisms, the presence of corrosion product could reduce local flexibilities, hence ultimately results in overall stiffened-up columns. The build-up of stresses within RC elements is referred to as “bursting stresses” in a recent report issued by International Federation for Structural Concrete (fib 2015). While the presence of such bursting stresses had not yet been specifically highlighted in the context of large-scale RC bridge piers, there are works confirming similar observations on smaller-scale or other types of RC specimens. As an example, an increase in normalized frequency is observed on moderately corroded RC beams, which is attributed to the increased friction at the steel-concrete interface as well as the continuous hydration of the cement compounds and silica fume (Maalej et al. 2010). Similarly, associated small increases in load capacity of RC beams have also been observed with low amounts of corrosion loss, owing to the increase of bond strength (fib 2000; Kallias and Imran Rafiq 2013). Similar responses were obtained from other sledgehammer blows in both x- and y-directions, as summarised in Table 3. On average, the first mode natural frequencies of specimens Column A1 and Column B1 increased by 10% and 23% respectively, following accelerated corrosion.

**Table 3:** Comparison of estimated first mode natural frequencies of column A1 and column B1, under free-standing condition, before and after processed by accelerated corrosion

Excitation direction	Measurement direction	Column A1			Column B1		
		Uncorroded	Corroded	Variation due to corrosion	Uncorroded	Corroded	Variation due to corrosion
x-axis	x-axis	8.75 Hz	9.50 Hz	+ 9%	11.75 Hz	14.50 Hz	+ 23%
	y-axis	8.75 Hz	9.50 Hz	+ 9%	11.75 Hz	14.50 Hz	+ 23%
y-axis	x-axis	8.50 Hz	9.25 Hz	+ 9%	11.75 Hz	14.75 Hz	+ 26%
	y-axis	8.50 Hz	9.75 Hz	+ 15%	12.00 Hz	14.50 Hz	+ 21%
Mean		8.63 Hz	9.50 Hz	+ 10%	11.81 Hz	14.56 Hz	+ 23%

#### Damping ratio

Estimations of damping ratio for the specimens Column A1 and Column B1, calculated using the half-power bandwidth method and the logarithmic decrement method, are summarised in Table 4. Results in uncorroded and corroded conditions are given separately, and the percentage change of damping ratio following corrosion is calculated. Using the half-power bandwidth method, the frequency domain estimations of the damping ratio for both specimens before corrosion are about 0.041, whereas the logarithmic decrement method yields time domain estimations of damping ratio of about 0.046. For Column B1, the two methods agreed well with each other, indicating a slight reduction in damping ratio (averaging 6%) as the result of corrosion. The outputs are relatively consistent between individual cases, i.e., between different sledgehammer testing scenarios and using two different estimation approaches. On the other hand, results from Column A1 have some scatter. The frequency domain estimation indicates that the damping ratio of Column A1 reduced by an average of 8% after the specimen was corroded, while the time domain approach suggests an increase of 10%. Contradictory results were obtained between sledgehammer tests where the impact forces were applied in the two perpendicular directions. Responses from the y-direction sledgehammer blows generally showed a considerable increase in damping ratio, in the order of 30%, whereas the x-direction sledgehammer tests indicated the opposite. One of the cases in Table 4 is shown as N/A; this is due to a significant overlapping of multiple closely located peaks in its power spectrum, such that the half-power bandwidth of the first mode response is unidentifiable. Overall, the averaged effect of corrosion on the damping ratios of both RC columns was insignificant, although the random variability of the data, especially among individual estimates of damping ratio for Column A1, should be noted.

**Table 4:** Comparison of estimated damping ratios for column A1 and column B1 before and after accelerated corrosion

Half-power bandwidth method (frequency domain estimation)							
Excitation direction	Measurement direction	Column A1			Column B1		
		Uncorroded	Corroded	Difference due to corrosion	Uncorroded	Corroded	Difference due to corrosion
x-axis	x-axis	0.046	0.031	− 33%	0.040	0.040	0%
	y-axis	0.045	0.033	− 27%	0.047	0.042	− 11%
y-axis	x-axis	0.041	0.056	+ 37%	0.044	0.038	− 14%
	y-axis	0.031	–	–	0.033	0.036	+ 9%
Mean		0.041	0.040	− 8%	0.041	0.039	− 4%
Coefficient of Variation		17%	35%	–	15%	7%	–
95% confidence margin		0.030 ~ 0.052	0.006 ~ 0.075	–	0.031 ~ 0.051	0.035 ~ 0.043	–

Logarithmic decrement method (time domain estimation)							
Excitation direction	Measurement direction	Column A1			Column B1		
		Uncorroded	Corroded	Difference due to corrosion	Uncorroded	Corroded	Difference due to corrosion
x-axis	x-axis	0.048	0.038	− 21%	0.048	0.040	− 17%
	y-axis	0.040	0.047	+ 18%	0.045	0.042	− 7%
y-axis	x-axis	0.051	0.061	+ 20%	0.045	0.043	− 4%
	y-axis	0.045	0.056	+ 24%	0.044	0.043	− 2%
Mean		0.046	0.051	+ 10%	0.046	0.042	− 8%
Coefficient of variation		10%	20%	–	4%	3%	–
95% confidence margin		0.039 ~ 0.054	0.034 ~ 0.067	–	0.043 ~ 0.048	0.040 ~ 0.044	–
Mean of two methods		0.043	0.046	+ 6%	0.043	0.041	− 6%

### Effects of quasi-static loading cycles with incremental displacement amplitudes on the dynamic properties of RC bridge columns

#### Natural frequency and secant stiffness

**Fig. 7:**
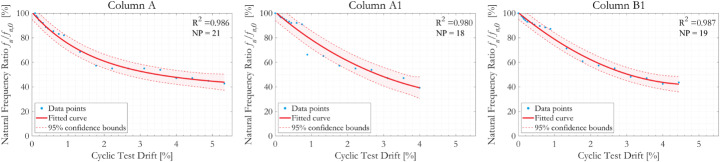
Evolution of normalised first mode natural frequency *f*_*n*_/*f*_*n,0*_ with column drift ratio during cyclic loading. *R*^*2*^ coefficient of determination, *NP* number of data points

Figure 7 presents the relationship between normalised first mode natural frequency *f*_*n*_/*f*_*n,0*_ and cyclic column drifts, where *f*_*n,0*_ is the as-built first mode natural frequency of the tested column specimens and *f*_*n*_ is the frequency measured during the sequence of loading cycles. Experimental measurements are plotted using blue markers and the red curves represent a fitted second order Gauss function to the data points and the associated 95% confidence bounds obtained from the regression analyses. The data points are weighted during the curve fitting processes force the fitted curves to pass through the initial data point, which has the physical meaning of zero degradation at zero column drift. The goodness-of-fit of the regressions is indexed by the coefficient of determination *R*^*2*^, marked in each subfigure. All three specimens were tested cyclically until significant distress appeared. On significant distress, all three specimens gave the similar residual natural frequency ratio *f*_*n*_/*f*_*n,0*_ of approximately 40% (i.e., an elongation of the natural period of 250%).

The effect of corrosion can be demonstrated by comparing responses from Column A and Column A1. Column A underwent exhibited a drift ratio of 5.3% before test termination and Column A1 just 4.0% (final displacement amplitudes were 96.0 mm and 72.0 mm, respectively). In contrast, the fitted curves for Columns A1 and B1 showed similar trends and terminated at similar drift magnitudes; this is in line with expectations, given that both columns exhibited similar degrees of corrosion on their respective critical cross-sections, and that the decrease in natural frequency is normalised. Measured data points from all three specimens showed a relatively slow reduction in natural frequency at the initiation of the cyclic test, followed by a sudden fall in *f*_*n*_/*f*_*n,0*_ after loading cycles involving 13.4–16.0 mm displacement, equivalent to 0.74% and 0.89% drift. This might be attributed to the onset of major concrete cracking or crushing, as will be confirmed numerically in later sections.

**Fig. 8:**
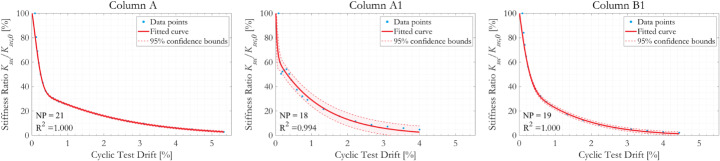
Evolution of normalised secant stiffness *K*_*sec*_/*K*_*sec,0*_ with column drift ratio during cyclic loading

**Fig. 9:**
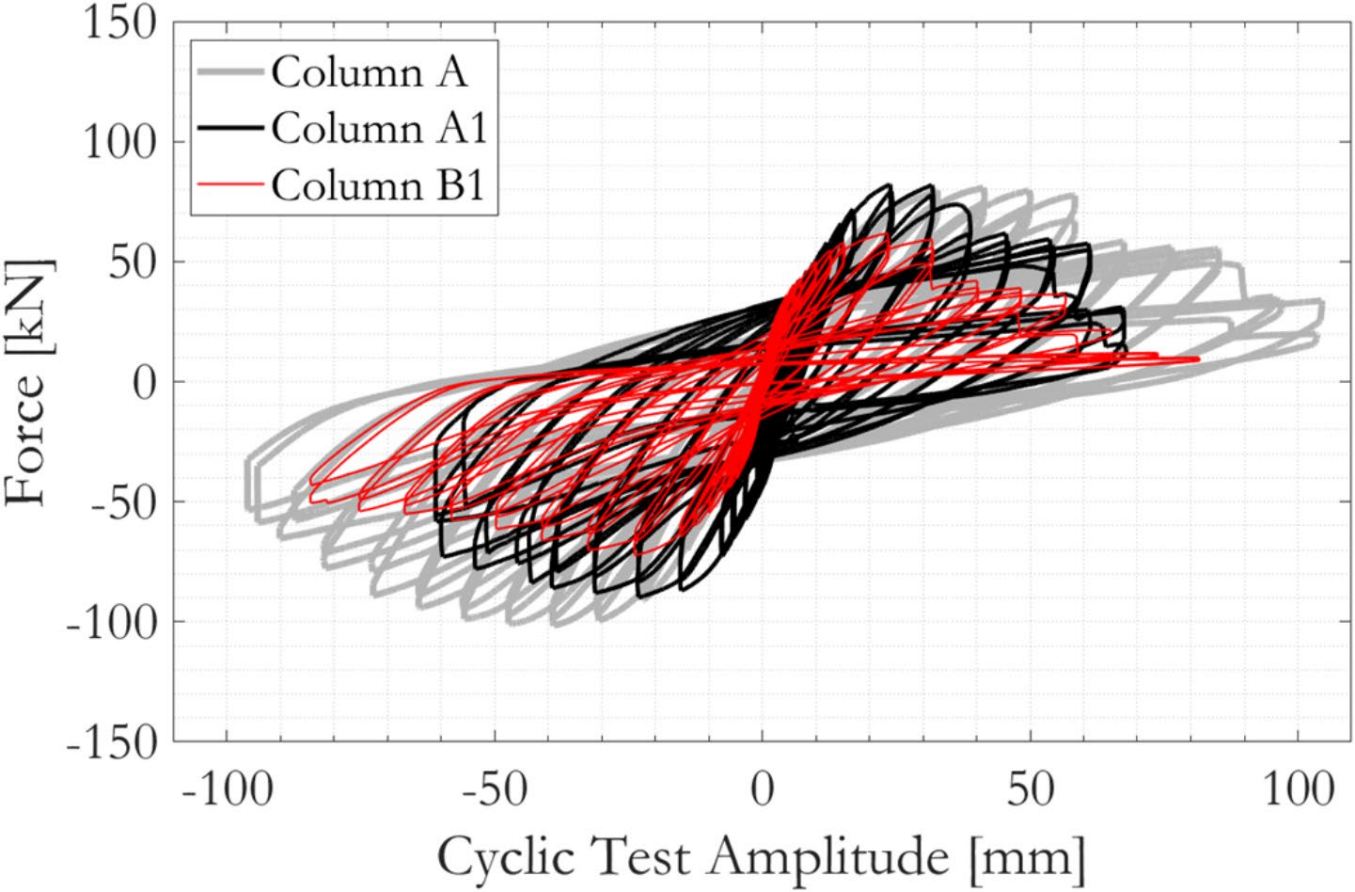
Force-displacement responses of the three specimens tested in cyclic loading, figure after (Kashani et al. 2024)

Figure 8 presents the evolution of normalised secant stiffness *K*_*sec*_/*K*_*sec,0*_ with drift during the sequence of cyclic loading. Despite the distinct differences between the force-displacement responses shown in Fig. 9, the trends in the normalised curves of *K*_*sec*_/*K*_*sec,0*_ against cyclic test drift for the three specimens are similar. The reduction in secant stiffness with increasing cyclic drift is characterised by two distinct stages; an initial rapid decrease in *K*_*sec*_/*K*_*sec,0*_ followed by a second stage in which the rate of stiffness degradation is much slower. The turning points of the two response stages are at around 0.5% column drift, or 9.0 mm displacement, although there are some anomalies in the data points obtained from the specimen Column A1. The goodness-of-fit of the data points to the fitted *K*_*sec*_/*K*_*sec,0*_ is generally high, with *R*^*2*^ values close to unity.

**Fig. 10:**
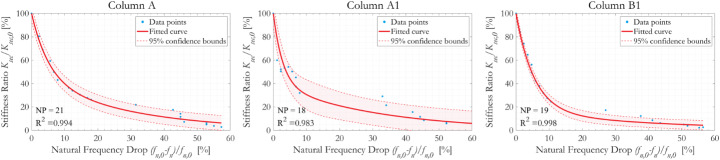
Relation between the normalised secant stiffness *K*_*sec*_/*K*_*sec,0*_ and normalised drop of first mode natural frequency (*f*_*n,0*_-*f*_*n*_)/*f*_*n,0*_, during cyclic tests with incremental displacement amplitudes

The above data can be correlated by the drift ratio during the cyclic test, with the percentage fall in the first mode natural frequency (*f*_*n,0*_-*f*_*n*_)/*f*_*n,0*_ used as an index to the residual secant stiffness *K*_*sec*_/*K*_*sec,0*_. The derived relations are shown in Fig. 10. The normalised *K*_*sec*_/*K*_*sec,0*_ versus (*f*_*n,0*_-*f*_*n*_)/*f*_*n,0*_ relations for all three specimens display strong nonlinearity. For the same fall in natural frequency (*f*_*n,0*_-*f*_*n*_)/*f*_*n,0*_, the residual secant stiffness *K*_*sec*_/*K*_*sec,0*_ is greatest for Column A and lowest for Column B1. Given their states of corrosion and the hoop reinforcement spacing, this is consistent with engineering judgement. Overall, a 5% reduction in column natural frequency from its initial state indicates a residual secant stiffness of 45–55%, and a 20% fall in the natural frequency corresponds to residual secant stiffness of 12–25% of the original.

**Fig. 11:**
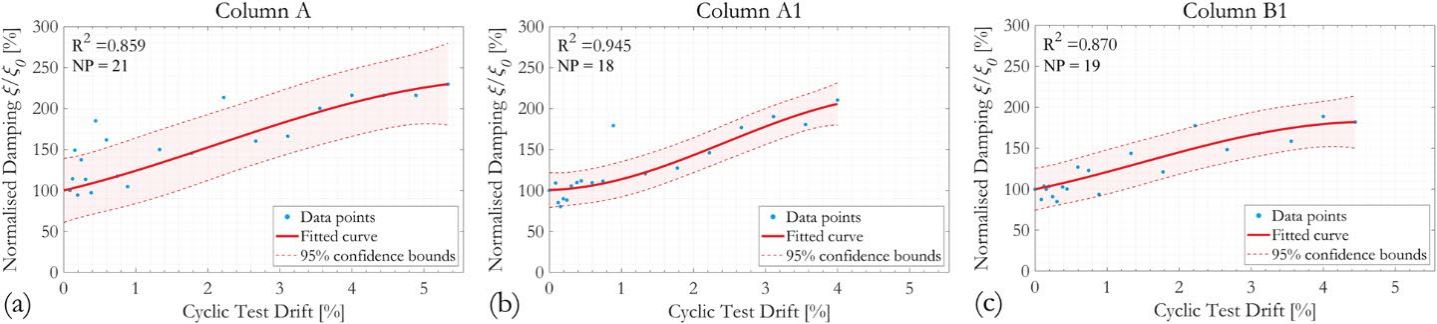
Evolution of normalised damping ratio *ξ*_*n*_/*ξ*_*n,0*_ with column drift ratio during cyclic loading, evaluated using half-power bandwidth method

**Fig. 12:**
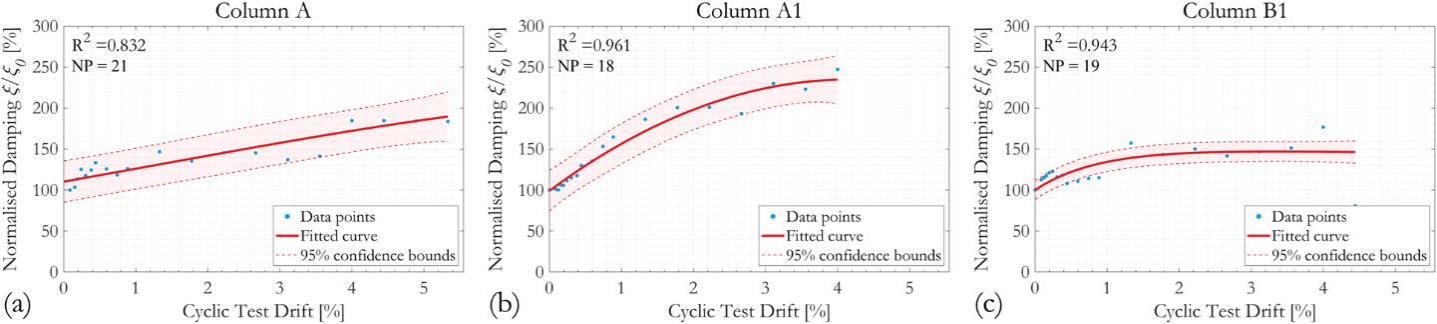
Evolution of normalised damping ratio *ξ*_*n*_/*ξ*_*n,0*_ with column drift ratio during cyclic loading, evaluated using logarithmic decrement method

#### Damping ratio

The evolution of normalised damping ratio *ξ*/*ξ*_*0*_ with column drift ratio during cyclic tests is shown in Fig. 11 using the half-power bandwidth method and Fig. 12 using the logarithmic decrement method. The damping ratios of all three RC column specimens tested increase as the cyclic test progresses, which agrees with previous observations from the literature, such as (Soleimani et al. 2021). The effect of corrosion can be observed by comparing results from specimens Column A and Column A1. Using the half-power bandwidth method, the effect of corrosion seems to be insignificant as the damping ratios of both specimens approximately doubled at 4% cyclic drift, whereas the logarithmic decrement method estimates the damping ratios of Columns A and A1 to be approximately 170% and 230% of the original respectively at 4% column drift. Scatter of the data points is more pronounced for Column A. The influence of the different hoop reinforcement spacing can be assessed by comparing results for Columns A1 and B1; the increase of damping ratio with cyclic drifts is less significant as a result of the grater hoop reinforcement spacing in specimen Column B1. Evolution of the equivalent viscous damping ratio of the RC columns was assessed graphically from their respective hysteresis loops, as in (Kashani et al. 2024).

4. Numerically calculated hysteresis response of cyclically loaded ageing rc bridge columns and the evolution of their properties.

### Development of nonlinear beam element fibre section model for RC columns

**Fig. 13:**
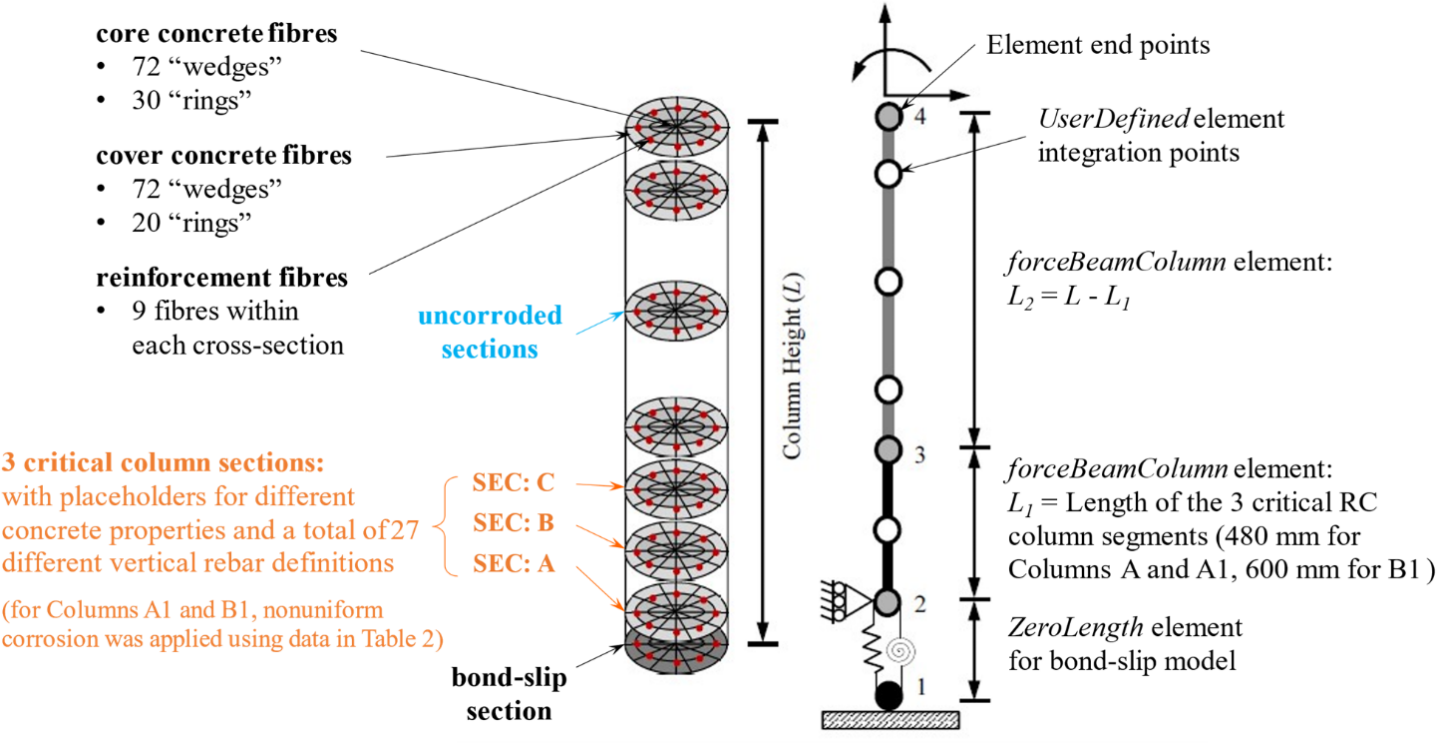
Overview of the nonlinear beam element, fibre section finite-element model for circular RC bridge columns. The 3 critical column sections (SEC: A, B, and C) correspond to those marked in Fig. 2. *L* = 1800 mm is the effective column length for all specimens

Numerical models of the tested RC columns, illustrated in Fig. 13, were developed using OpenSees v3.5.0 (McKenna 2011). The models are based on those documented in detail in (Kashani et al. 2016a, b; Dizaj et al. 2018) and the present work add the capability to simulate nonuniform material and geometric deterioration of critical reinforcement and concrete segments. The basis of the model, such as geometry and material properties, follows those of the tested columns (Table 1). Various model features used in the work are discussed in sections below and are also summarised in Table 5. Simulated responses were then compared with the experimental data outlined above to provide insights into the response mechanisms of RC columns under cyclic load. An adaptive solution script for OpenSees published in (Dong 2020) was used to improve numerical convergence.

**Table 5:** Summary of opensees model features used in this study

Overall model configuration	The model used is an extended version of those documented at length in (Kashani et al. 2016a, b; Dizaj et al. 2018), with the additional capability to capture nonuniform geometric-material degradation of reinforcement bars and concrete within critical column sections as shown in Fig. 13.
Geometries and Materials	All geometry and material properties used in the numerical models correspond to those from the experiments as in Table 1.
	*Hysteretic* and *Fatigue* uniaxial material models for steel reinforcement along the length of the column. (*Fatigue* material only applied in the corroded portion, that is, SEC A, B and C as shown in Fig. 13)
	*Steel02* uniaxial material model for reinforcement bond-slip at column base, with non-corroded material properties.
	*Concrete04* uniaxial material for confined and unconfined concrete along the length of the column.
	*Concrete01* uniaxial material model for the base section, with non-corroded material properties.
Elements	*ZeroLength* element with *fibre* section for bond-slip at column base.
	*forceBeamColumn* with *fibre* section and *UserDefined* integration scheme along the length of each column.
Model features and calculations	Modelling of vertical reinforcement buckling, including estimating the nonlinear compressive stress-strain relations and the buckling lengths, follows (Dhakal and Maekawa 2002).
	Modelling of reinforcement property deterioration owing to corrosion follows the relation proposed in (Du et al. 2005a, b) and the selection of an associated coefficient *p* for various properties follows (Kashani et al. 2013b; Dizaj et al. 2021). The elastic flexural rigidity of column is modified for the nonlinear buckling following (Kashani et al. 2016b).
	Modelling of reinforcement hysteresis pinching characteristics follows (Kashani et al. 2016a) and modifications accounting for corrosion follows (Dizaj et al. 2018).
	Calculation of core and cover concrete model parameters follows (Scott et al. 1982; Paulay and Priestly 1992; Dizaj et al. 2018)
	Modelling of corrosion induced cracking and spalling on cover concrete follows (Coronelli and Gambarova 2004)
	Calculation of the rebar stress-slip relationship at the base of the RC column specimens follows (Lowes and Altoontash 2003).
	Reinforcement bar low-cycle fatigue parameters selected following (Kashani et al. 2015b).

#### Definition of finite elements and the bond-slip model

The finite-element model of the RC bridge columns comprised three elements; from bottom to top, one *ZeroLength* element for simulating the bond-slip response of the column at its base, one *forceBeamColumn* element covering the length of the three critically corroded cross-sections, and a second *forceBeamColumn* element accounting for the remainder upper portion of the RC columns.

A zero-length section element was used to model the strain penetration and the slippage of vertical reinforcement bars anchored to the foundation (Berry and Eberhard 2003). This can be triggered by the formation of plastic hinges during lateral cyclic loading and has been previously observed experimentally on RC bridge columns (Lehman and Moehle 2000). In this study, the bond-slip model for simulating end slip of longitudinal reinforcements in beam-column joints developed by Lowes and Altoontash (Lowes and Altoontash 2003) was used to calculate the rebar stress-slip relationship at the base of the RC column specimens. Uncorroded material properties were used, given that the reinforcements are deeply anchored into the foundation block hence well protected against corrosion. Implementation of the bond-slip model in OpenSees utilised the uniaxial material model *Concrete01* for the concrete and *Steel02* (Filippou et al. 1983) for the steel reinforcements.

Force-based finite-elements were adopted as they give more accurate responses then their displacement-based counterparts when analysing the nonlinear cyclic behaviour of RC columns (Coleman and Spacone 2001; Correia et al. 2008). The length of the first (lower) force-based beam-column element was 480 mm for Columns A and A1 and 600 mm for Column B1, covering the three critical column sections near the base of each specimen (Fig. 2). The *UserDefined* element integration scheme (Scott 2011) was used, with three integration points evenly distributed along the element weighted 0.167:0.666:0.167. Cross-sectional properties of the critical column sections A, B, and C respectively were assigned to the three integration points. This element setup was found to produce the hysteresis loops best matched to the experimental results, and also numerically stable for all three column configurations with cross-sections having either different or identical properties. This is not otherwise achieved when attempting to represent the three critical column segments using either a single beam-column element with more than three integration points or multiple force-based beam-column elements, regardless of the number of integration points within each. Adopting this element setup means that the element integration length is slightly longer than the analytically calculated rebar buckling length (Dhakal and Maekawa 2002) and does not match an integer multiple of the hoop rebar spacing. Nonetheless, the integration length of the first element is still close to the experimentally observed buckling length of the tested specimens, of approximately 160 mm for Columns A and A1 and 200 mm for Column B1. It is acknowledged that the slightly overestimated rebar buckling lengths tend to give conservative numerical results regarding the low-cycle fatigue life of vertical steel reinforcements (Kashani et al. 2015b).

For the second (upper) force-based beam-column element, the *UserDefined* integration scheme with five integration points was used, with uncorroded cross-sectional properties. The integration points are located at fractions of the element length of 0.0, 0.1727, 0.5, 0.8273 and 1.0, weighted 0.05:0.2722:0.3556:0.2722:0.05. A convergence analysis determined the optimal number of subdivisions in the circumferential direction of each fibre cross-section (number of wedges) to be 72 and the optimal number of subdivisions in the radial direction (number of rings) to be 50. These values were found to achieve consistent results with a minimum number of fibres, while avoiding numerical instability.

#### Corrosion induced nonuniform deterioration of concrete

Unconfined and confined concrete were modelled in OpenSees using the *Concrete04* material model (Mander et al. 1988). Model parameters for confined and unconfined concrete, including the confinement factor, the maximum stress factor, the post-peak softening slope, maximum stress and strain, were calculated as in (Scott et al. 1982). For the artificially corroded specimens A1 and B1, degraded concrete properties were further determined using the modified Mander model (Dizaj et al. 2018) and implemented to simulate the column responses to lateral cyclic loading. When corrosion of hoop reinforcements starts, the cross-sectional area, yielding strength and fracture strain of ties can decrease over time. Reduction in these parameters were determined by correlating to a factored corrosion mass loss following (Dizaj et al. 2018). The compressive strain of confined concrete at the instance of hoop reinforcement fracturing was calculated according to (Paulay and Priestly 1992) as a function of compressive strength of the core concrete and the strain at maximum stress of uncorroded transverse reinforcement steel.

The effect of corrosion induced cracking and spalling on cover concrete was accounted for using compression field theory as described in (Coronelli and Gambarova 2004). This is achieved by introducing a reduction in strength for the cover concrete and brittle post-peak behaviour. In general, the characteristic crack width induced by each rebar was first calculated based on an empirically estimated pitting depth and the proportional volumetric expansion of the corrosion product, assuming that all corrosion products accumulate around the corroded rebar. Reduction of the compressive strength was then calculated depending on the average tensile strain in the transverse direction of the column sections, evaluated as the ratio of total crack width to the perimeter of the uncorroded column section.

**Fig. 14:**
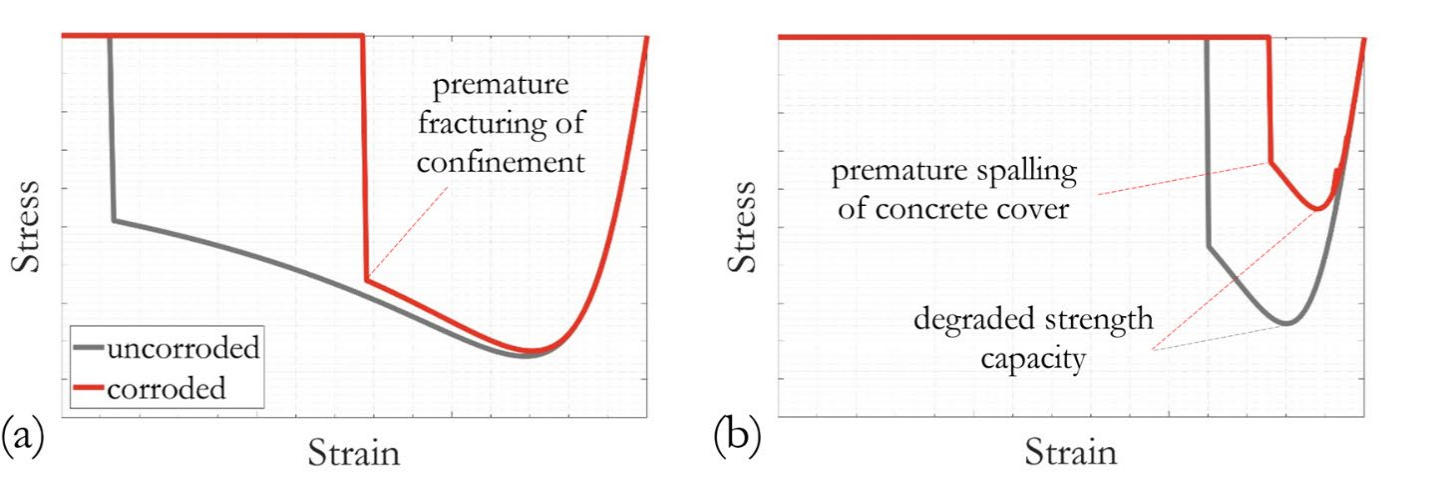
Schematics of typical stress-strain models for uncorroded and corroded concrete: **a** confined and **b** unconfined

A schematic comparison of the simulated stress-strain responses of uncorroded and corroded concrete materials are given in Fig. 14. Within the three critical column segments near the bottom of each specimen, a total of 6 sets of modified concrete material parameters were calculated and assigned separately, accounting for nonuniformity of corrosion along the RC column as well as the differences between confined and unconfined areas. The confinement effect from hoop reinforcements and its deterioration due to corrosion were accounted for implicitly in material properties of confined concrete.

#### Corrosion induced nonuniform deterioration of reinforcements accounting for buckling, fracturing, and low-cycle fatigue

Corrosion induced material mass losses from the steel reinforcing bars were accounted for by applying equivalent reductions to the rebar diameter, which were modelled in OpenSees as individual *fibres*. The nonuniform nature of corrosion mass losses along the vertical reinforcement was accounted for by assigning different corroded fibre cross-sectional areas (using direct experimental observations as in Table 2) to the 27 rebar segments distributed within the three critical RC column segments (as in Fig. 2).

A total of 27 sets of corrosion-deteriorated steel material properties were calculated and applied accordingly. The residual yield stress, ultimate stress and fracture strain of corroded vertical reinforcements were calculated using the empirical Eq. (4) suggested in (Du et al. 2005a, b), as a function of averaged corrosion mass losses for each individual rebar segment:

4 $$\:{Q}_{corr}=Q\left(1-p\psi\:\right)$$

where *Q* and *Q*_*corr*_ are the abovementioned quantities related respectively to uncorroded and corroded vertical reinforcements; *ψ* is the segmental rebar mass loss; and *p* is a pitting coefficient, taken as 0.005 for yield and ultimate stresses, and 0.035 for fracture strain (Dizaj et al. 2021). Subsequently, the nonlinear compressive stress-strain relation of each corroded rebar segment was determined using the Dhakal and Maekawa buckling model (Dhakal and Maekawa 2002), which defines their post-buckling responses. The combined use of formulation in Eq. (4) and the selection of the abovementioned pitting coefficient also considers the impact of geometric nonlinearity on the cyclic behaviour of corroded reinforcement bars (Kashani et al. 2015c). Buckling of reinforcement bars is an inelastic phenomenon, hence the elastic flexural rigidity is no longer valid. A modified flexural rigidity that has been validated against an extensive set of experimental data (Kashani et al. 2016b) was employed herein, accounting for the altered second moment of area and yield strength of corroded reinforcement. Corrosion does not have any influence on the elastic modulus of steel.

**Fig. 15:**
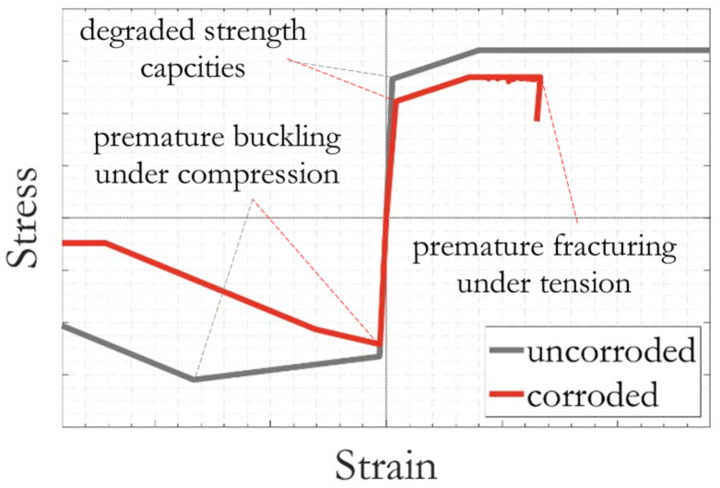
Schematics of the typical stress-strain model for uncorroded and corroded reinforcement steel

Implementation in OpenSees was achieved using the uniaxial material *Hysteretic*. To avoid strain localisation due to the post buckling response of reinforcements, the integration length of finite element near the base of the columns should ideally be equal to the buckling length (Kashani 2024). In the present model, the integration length was set to be slightly larger than the calculated buckling length (Dhakal and Maekawa 2002) to facilitate the modelling of the three critical column segments (Fig. 2). Figure 15 gives a schematic comparison of the simulated stress-strain responses of uncorroded and corroded reinforcement steel. The hysteresis responses of vertical reinforcements are typically pinched, with the shape affected by the stiffness of the hoop reinforcements. This was accounted for by assigning reloading factors of 0.4 and 0.6 for strain and stress respectively, following the recommendation obtained from a comprehensive parametric study in (Kashani et al. 2016a).

The low-cycle fatigue degradation of vertical reinforcing bars was modelled using the OpenSees uniaxial material *Fatigue* (Ballio and Castiglioni 1995). The *Fatigue* material works in conjunction with the *Hysteretic* material model, which does not change the stress-strain behaviour of the latter but keeps track of strain amplitudes using a modified rain-flow cycle counter (Uriz 2005) and the Miner’s Rule (Miner 1945). The onset of fatigue failure on corroded reinforcements was considered using a modification, described in (Kashani et al. 2013b), of the well-known Coffin-Manson relationship (Coffin 1954; Manson 1965), which relates the total strain amplitude to fatigue life. The degree of corrosion damage has an impact on the number of half-cycles to failure (Kashani et al. 2015a), which has been shown to depend on the reinforcement slenderness ratio. Values of fatigue parameters (i.e., strain at which one cycle will cause failure, *E0*, and the slope of the Coffin-Manson fatigue curve on a log-log plot, *m*) were assigned to each individual rebar segment following recommendations in (Kashani et al. 2015b), given as a function of vertical reinforcement slenderness ratio. For Columns A and A1 in which slenderness ratio is 5, *E0* and *m* were taken as 0.138 and − 0.393; for Column B1 in which slenderness ratio is 12.5, *E0* and *m* were taken as 0.257 and − 0.677. The effect of corrosion was included by updating the fatigue parameter *m* using Eq. (4), with the pitting coefficient *p* taken as −0.004 (Kashani et al. 2013b). In addition, the calculated fracture strain of corroded rebars were incorporated within the *Fatigue* uniaxial material too, so that reinforcement fracturing could also be triggered by a single event of strain exceedance.

### Responses of validated numerical models

#### Rebar and concrete responses

**Fig. 16:**
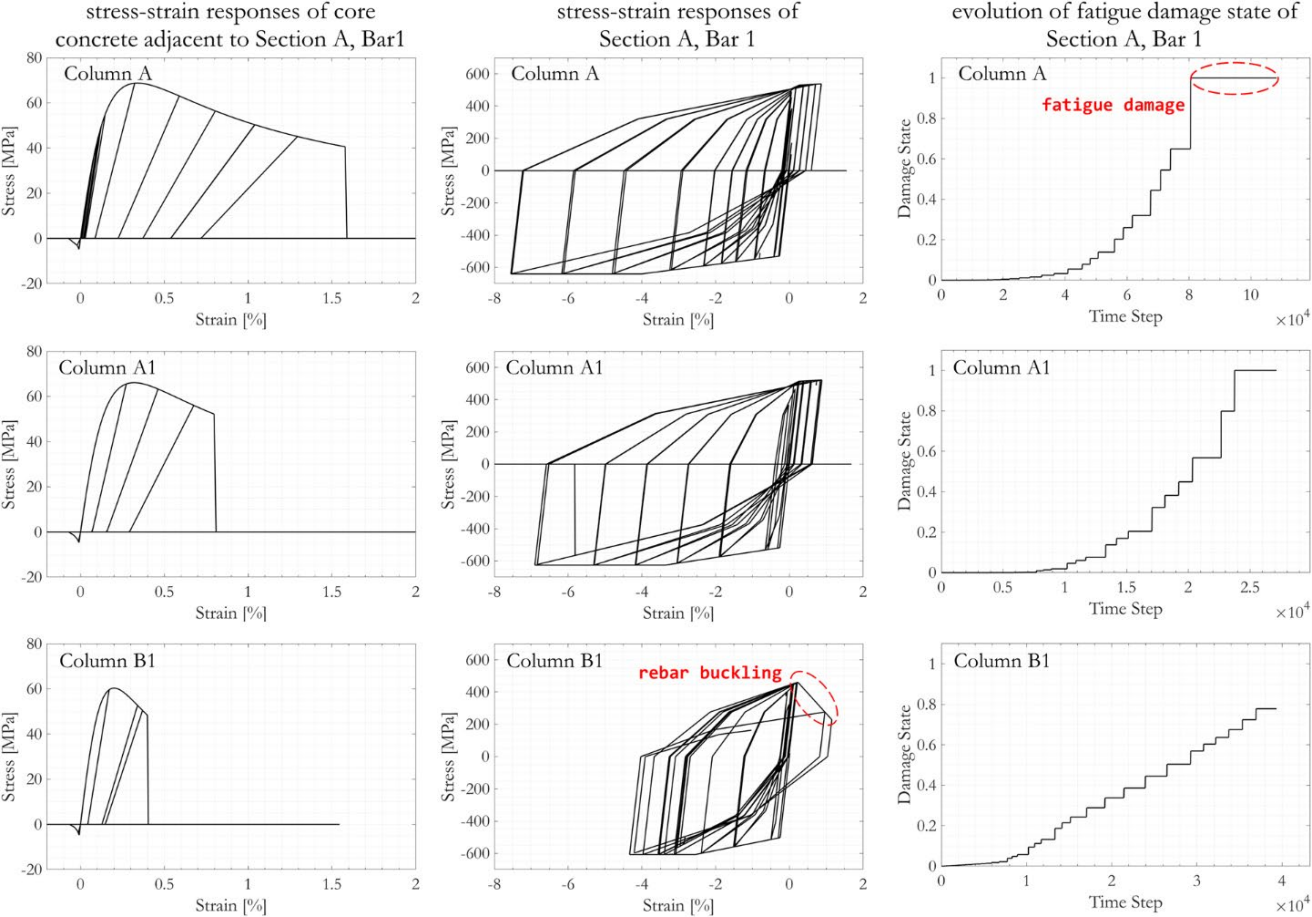
Examples of simulated core concrete and steel reinforcement fibre responses to cyclic loading, for Columns A, A1, and B1

Figure 16 shows exemplar results from the nonlinear fibre section, beam element OpenSees model for Columns A, A1 and B1. The location considered is the vertical reinforcement bar 1 and the adjacent core concrete (as indicated in Fig. 3b) on Section A (location as indicated in Fig. 2b). The cause of ultimate failure in Column A and A1 was found to be fatigue damage to the rebars, whereas in Column B1 the rebars experienced buckling rather than fatigue owing to the higher overall corrosion mass loss.

The core concrete in both specimens withstood tensile stress during the first level of cyclic loading displacement amplitude of 1.6 mm, and was found to be cracked after the first loading cycle of amplitude 2.25 mm (0.13% column drift ratio). After this, the tensile stress in the core concrete closest to the location of vertical reinforcement bar 1 remained at zero. In compression, taking Column A1 as an example, the core concrete in the immediate vicinity of bar 1 crushed during the first loading cycle of amplitude 48 mm (2.7% column drift) loading cycle, while the cover concrete fibre in the immediate vicinity of bar 1 crushed after the 13.4 mm (0.74% drift) loading cycles. The level of corrosion had a strong impact on the concrete strain at crushing, which was 1.6% for the uncorroded Column A, 0.8% for the moderately corroded Column A1, and 0.4% for the more severely corroded and less confined Column B1.

Regarding the vertical steel reinforcement, again taking Column A1 as an example, bar 1 was found not to be loaded in compression until completion of first loading cycle of displacement amplitude 10.7 mm (0.59% drift), as a result of the gradual deterioration of the concrete during cyclic loading and the consequent redistribution of stresses. As one of the bars furthest from the neutral axis under the applied lateral load, bar 1 was found to have yielded after the first loading cycle of displacement amplitude 13.4 mm (0.74%), and to have fractured at a displacement of 64.0 mm (3.6% drift) due to low-cycle fatigue. The impact of corrosion on the stress capacity of reinforcements are minor. Tensile strain development on reinforcement for Column B1 was noticeably limited, likely due to the combined effect of corrosion induced material and sectional deterioration and early concrete crushing. Fatigue damage of the rebar can be confirmed by inspecting the evolution of the rebar fatigue state. When the value of the fatigue state parameter is at or greater than 1, the rebar is considered numerically to have failed and no longer able to sustain any stress. Stress-strain curves for bar 1 and the adjacent core concrete show higher strains at zero stress, indicating that the numerical analysis was able to converge and continue even after the initiation of core concrete crushing or bar 1 fracturing.

#### Evolution of column stiffnesses and natural frequencies

**Fig. 17:**
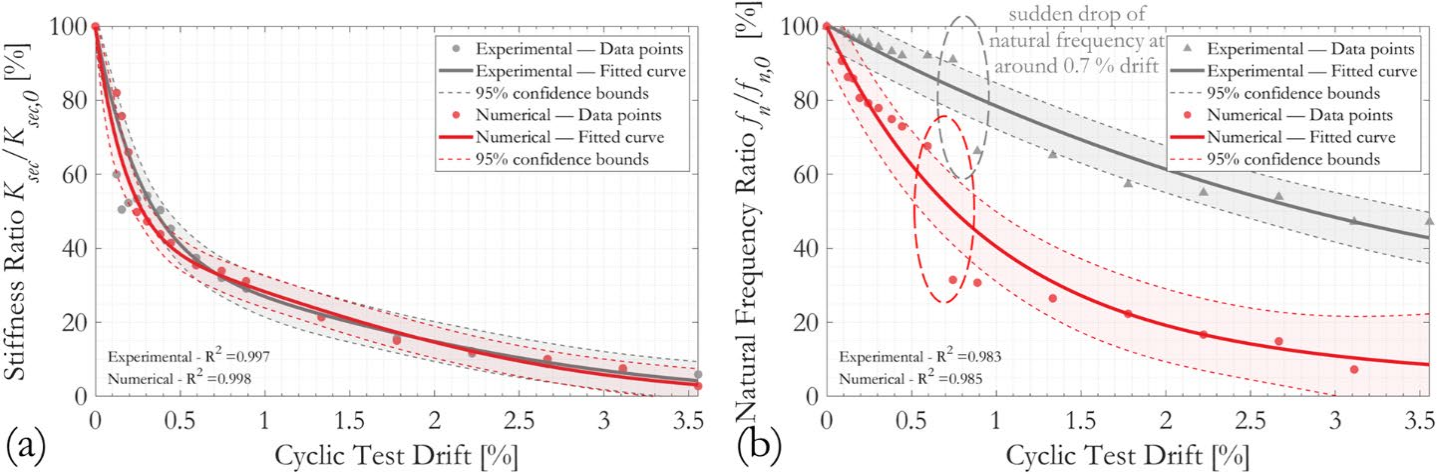
Comparison of Column A1 experimental and numerical results of the evolution of **a** normalised secant stiffness *K*_*sec*_/*K*_*sec,0*_ and **b** normalised first mode natural frequency *f*_*n*_/*f*_*n,0*_ with column drift ratio during cyclic loading

Comparison of the experimental and numerical responses of the normalised secant stiffness *K*_*sec*_/*K*_*sec,0*_ and normalised first mode natural frequency *f*_*n*_/*f*_*n,0*_ with increasing cyclic drift in Fig. 17 for Column A1 shows a generally close match. The simulated response captures the magnitude of the residual column stiffness as well as the two-stage degrading process; an initially high rate of degradation followed by a slower stage, with the turning point at about 0.5% column drift or 9 mm column-top displacement. Detailed inspection of the fibre responses at Section A of Column A1 shows that the cyclic drift at the turning point corresponds approximately to the moment when the cyclic compressive forces on both sides of the neutral axis are no longer taken solely by the concrete but are shared by the vertical steel reinforcements too. The gradual but evident change of the rate of column stiffness reduction, therefore, seems to reflect the degradation of the cover and core concrete during the repeated loading cycles. The fitted *K*_*sec*_/*K*_*sec,0*_ curves also generally align well with the data points, showing a high goodness-of-fit to the experimental results.

On the other hand, despite having the same reducing trend, the calculated evolution of normalised first mode natural frequency *f*_*n*_/*f*_*n,0*_ with cyclic drift does not match with the experiment in terms of magnitude. The experimental testing protocol dictates that a matching numerical simulation would require a sequence of consecutive and staggered static and modal analyses; and with the increasing cyclic amplitudes and potentially the ongoing development of nonlinearity, it is apparent that the natural frequencies estimated at all subsequent states are not entirely realistic. Nonetheless, the numerical simulation does capture the overall reducing trend as well as the sudden natural frequency drop occurred experimentally after completion of the 13.4 mm (0.74% column drift) loading cycles. The fact that these features are indeed captured, at least qualitatively, is evidence showing that the model is capable of simulating physics-based column failure mechanisms via its fibre discretised cross-sections. Inspection of individual fibre responses indicates that this sudden change in column modal characteristics is related to the onset of cover concrete spalling and vertical rebar yielding. The trends observed in the numerical models for Column A and Column B1 are similar.

#### Validated force-displacement responses

**Fig. 18:**
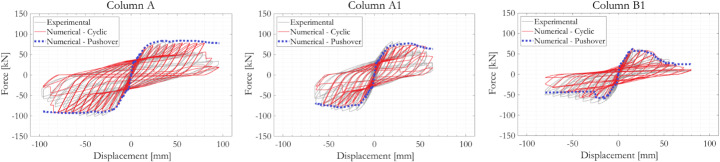
Comparison of experimentally measured and numerically simulated force-displacement responses of the three RC column specimens. Numerically simulated pushover responses are overlayed

Figure 18 compares experimentally measured and numerically simulated column-specific force-displacement responses. The OpenSees representations of the three tested RC column specimens, which have distinctly different responses, are able to reproduce well not only the force amplitudes at each loading cycle but also the shape of the hysteresis loops including the pinching effect. The numerical models are also capable of reproducing, both in magnitude and timing, the multiple sudden drops in force at large displacements, which occur as a result of areas of core concrete crushing and the initiation of fatigue damage of individual vertical reinforcement bars. The numerical analyses aborted at the same cyclic loading amplitude as structural failure occurred in the experiments on Column A and B1, and, for Column A1, one level of displacement amplitude earlier. The numerically simulated force-displacement relations have zero slope with near-zero forces on the final loading cycles before the finite-element analyses abort. This is believed to be a numerical anomaly and this final segment of problematic data, which occurs well after the onset of core concrete crushing or vertical reinforcement bar fracture, can be discarded. Physics-based local column failure mechanisms (as reflected in Fig. 16) can be traced from the overall hysteresis responses. For Columns A and A1, the dictating response mechanism associated with the overall column strength drop towards their last cyclic loading amplitude is the initiation of fatigue damage on some of the vertical rebars. For Columns B1, a sudden drop in overall column strength at the cyclic loading amplitude of around 40 mm can be associated with the exceedance of crushing strain at some part of core concrete and the subsequent stress drop on nearby vertical rebars, suggesting the initiation of vertical rebar buckling followed by core concrete loss. These simulated failure processes agreed qualitatively with the experimental observations.

Pushover responses of the three columns in both directions are overlayed on the force-displacement plots as blue dotted curves. The pushover curve generally matches but us not always identical to the envelope of the column hysteresis loops. For Columns A and A1, no sudden drops are observed in the pushover results. The columns can sustain almost constant force at and even beyond the lateral displacement amplitude at which the same column would have failed if loaded cyclically. For Column B1, early fracture of a rebar accompanied by a sudden drop of force on the column’s pushover curve occurred in the negative direction, before this happened on the same column subjected to cyclic loading. While it cannot be confirmed, this is likely to be attributable to numerical issues related to the selection and configuration of material models and finite-elements in OpenSees. In any case, it reflects a fair level of uncertainty when attempting to reproduce the cyclic responses of RC columns numerically. Overall, the agreement between the numerical pushover results and the envelope to the cyclic responses is satisfactory, hence showing the validated OpenSees model to be a useful tool for future studies of circular RC bridge piers.

**Fig. 19:**
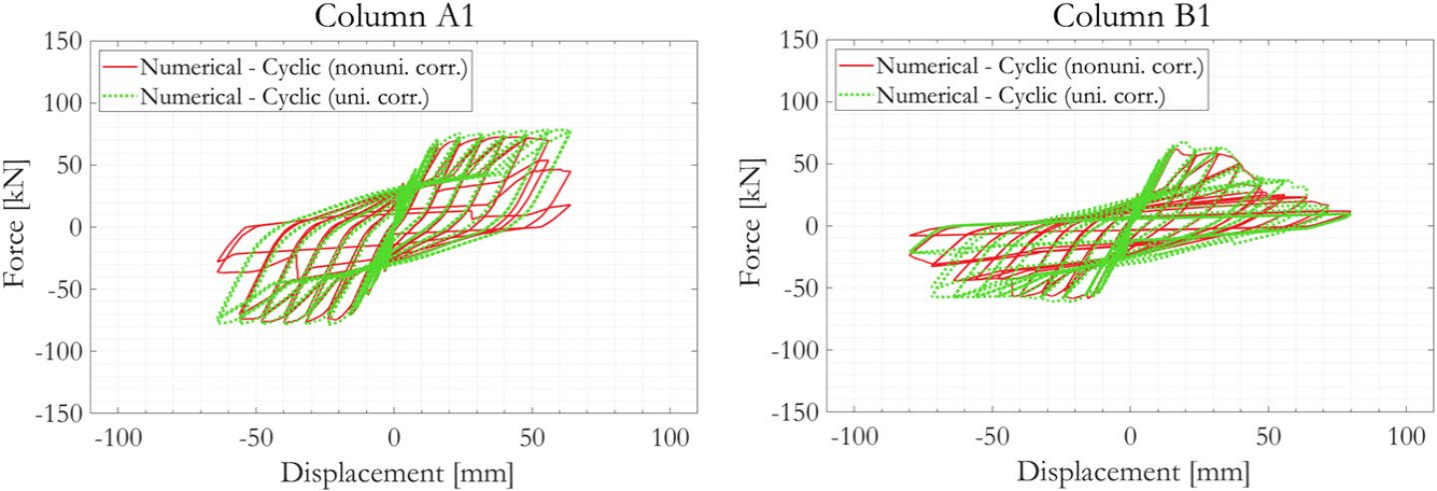
Comparison of numerically simulated force-displacement responses of the two corroded RC column specimens considering uniform or nonuniform material-geometric deterioration

The effect of the explicit modelling of nonuniform material-geometric degradation in Columns A1 and B1 is shown in Fig. 19. For column A1, bar 1 and 2 in Section A are corroded considerably more than all the rest of the rebar segments (Table 2). When the averaged rebar corrosion mass loss value (6.59%) is applied throughout the column, all vertical rebar segments never experienced fatigue damage, buckling or fracturing. This is reflected in the overall hysteresis loop by the fact that no degradation of overall column strength occurred up to cyclic loading amplitude of 64 mm, at which both the experimental observation and the numerical simulation considering nonuniform degradation suggested significant column distress. For Column B1, the differences between the hysteresis loops are less pronounced. This is because even when then applied corrosion mass losses are uniform (13.99%), its magnitude is still sufficiently large to trigger various failure mechanisms at just slightly larger cyclic loading amplitudes. These observations indicate the possibility of overestimating structural capacity if corrosion nonuniformity is neglected in structural assessments (reflected herein by the delayed sudden column strength drops from the 56 mm to the 64 mm loading cycle for Column A, and from the 48 mm to the 64 mm loading cycle for Column B). On the other hand, a scenario in which the capacity could be underestimated by neglecting corrosion nonuniformity has not been observed and is considered less probable. The discrepancies also point to the likely sensitivity of severely corroded column responses to its position (around its vertical centroid axis) relative to input lateral load, considering the probable anisotropy of residual lateral resistance owing to nonuniform corrosion. As an example, a premature or a delayed vertical rebar buckling could be possible if the worst-corroded vertical rebar on a column is positioned furthest away from or closet to the neutral axis of the input lateral load.

## Concluding remarks

In this paper, the response and the evolution of dynamic properties of three large-scale circular RC column specimens subjected to repeated loading cycles have been investigated by means of laboratory cyclic and sledgehammer testing. Various experimentally measured responses from the tests were subsequently used to develop and validate an advanced nonlinear beam element, fibre section finite-element model using OpenSees for simulating the degradation of new and corroded circular RC bridge piers subjected to cyclic loading.

An experimental campaign involved the determination of initial modal properties of the three differently configured RC column specimens, before two of them were artificially corroded to replicate ageing effects. The specimens were then subjected to quasi-static loading cycles with of increasing displacement amplitude, between each of which sledgehammer tests were carried out. The measured data were then processed in both the time and frequency domains to identify the evolution of lateral secant stiffness, first mode natural frequency, and damping ratio. Key experimental findings are as follows.

- When subjected to corrosion, the first mode natural frequency of the tested columns increased by 10–23%, whereas the change in damping ratio showed statistically large variation but was on average insignificant.
- Under cyclic loading, the normalised first mode natural frequency *f*_*n*_/*f*_*n,0*_ of the RC columns reduced to approximately 40% upon significant column distress. The effect of heavy corrosion weakened the column such that the corresponding column drift ratio reduced from 5.3 to 4.0%.
- The relation between the normalised residual lateral secant stiffness *K*_*sec*_/*K*_*sec,0*_ and the normalised drop in measured natural frequency (*f*_*n,0*_-*f*_*n*_)/*f*_*n,0*_ provides useful information for the rapid structural assessment of RC bridge columns after a seismic event. A 5% reduction in first mode natural frequency from its original value corresponds to system residual stiffness of 45–55%; and a 20% drop in first mode natural frequency to a residual stiffness of 12–25%.
- On average, the normalised damping ratio *ξ*/*ξ*_*0*_ of the RC columns subjected to cyclic loading increased to approximately 200% on significant column distress.

A computationally efficient, nonlinear beam element, fibre section finite-element model was developed, able to account for the geometrical and mechanical deterioration of steel reinforcement due to corrosion. The nonuniform nature of this deterioration is captured by the model for individual rebar segments located in three critically corroded and heavily loaded cross-sections at the base of the RC columns. The model also simulates the precise as-constructed locations of vertical reinforcement bars and the effects of inelastic buckling, low-cycle fatigue, bond-slip and rebar fracture. Key findings from the application of the model are as follows.

- The mean and statistical variability of numerically calculated relations between *K*_*sec*_/*K*_*sec,0*_ and cyclic drift match well the experimental observations. Examination of stress-strain responses for individual numerical fibres shows that the distinctive change in stiffness reduction rate at about 0.5% drift can be attributed to the degradation of concrete and the resulting redistribution of compression loads between concrete and steel reinforcement that occurs and accumulates before the turning point.
- The numerically simulated *f*_*n*_/*f*_*n,0*_ versus cyclic drift relations do not match the experimental results in terms of absolute values. Nonetheless, the trend in relative change of individual data points as well as the sudden natural frequency drop at approximately 0.7% drift were both reflected. The latter was found to correspond to the onset of cover concrete crushing and vertical reinforcement yield.
- Necessity of the explicit modelling of nonuniform geometric-material degradation is demonstrated by a comparison of overall hysteresis loop as well as localised fibre-section responses. The numerically calculated force-displacement responses of the three tested RC columns reflect well the force amplitudes at each loading cycle, the pinched shape of the hysteresis loops, and the multiple sudden drops in force at large displacements due to areas of core concrete crushing and fatigue induced fracture of vertical reinforcement bars. The proposed OpenSees finite-element model is able to capture the responses of all three differently configured RC bridge column specimens tested, without making column-specific adjustments.
